# Development of Bioluminescent Bioreporters for *In Vitro* and *In Vivo* Tracking of *Yersinia pestis*

**DOI:** 10.1371/journal.pone.0047123

**Published:** 2012-10-11

**Authors:** Yanwen Sun, Michael G. Connor, Jarrod M. Pennington, Matthew B. Lawrenz

**Affiliations:** Center for Predictive Medicine for Biodefense and Emerging Infectious Diseases, Department of Microbiology and Immunology, University of Louisville School of Medicine, Louisville, Kentucky, United States of America; Cornell University, United States of America

## Abstract

*Yersinia pestis* causes an acute infection known as the plague. Conventional techniques to enumerate *Y. pestis* can be labor intensive and do not lend themselves to high throughput assays. In contrast, bioluminescent bioreporters produce light that can be detected using plate readers or optical imaging platforms to monitor bacterial populations as a function of luminescence. Here, we describe the development of two *Y. pestis* chromosomal-based *luxCDABE* bioreporters, Lux_P*tolC*_ and Lux_P*cysZK*_. These bioreporters use constitutive promoters to drive expression of *luxCDABE* that allow for sensitive detection of bacteria via bioluminescence *in vitro*. Importantly, both bioreporters demonstrate a direct correlation between bacterial numbers and bioluminescence, which allows for bioluminescence to be used to compare bacterial numbers. We demonstrate the use of these bioreporters to test antimicrobial inhibitors (Lux_P*tolC*_) and monitor intracellular survival (Lux_P*tolC*_ and Lux_P*cysZK*_) in vitro. Furthermore, we show that *Y. pestis* infection of the mouse model can be monitored using whole animal optical imaging in real time. Using optical imaging, we observed *Y. pestis* dissemination and differentiated between virulence phenotypes in live animals via bioluminescence. Finally, we demonstrate that whole animal optical imaging can identify unexpected colonization patterns in mutant-infected animals.

## Introduction

Bioreporters are engineered microbes that produce a detectable signal that can be used to monitor cell populations or responses to environmental stimuli. The bacterial *luxCDABE* operon, which produces light through bioluminescence, has been adapted for use as a bioreporter in many species of bacteria [1]. Unlike eukaryotic luciferase systems, the *luxCDABE* operon produces both the luciferase enzyme and the substrates required for light production, removing the requirement for supplemental exogenous substrates for luminescence [2]. By replacing the native *luxCDABE* promoter with a promoter from a gene of interest, researchers can monitor changes in gene expression as a function of bioluminescence. *luxCDABE* reporters driven by constitutive promoters, in which bacterial density directly correlates to luminescence, provide a system to monitor bacterial growth. Furthermore, because bioluminescence is only produced by viable bacteria, bacterial survival can also be monitored with a *luxCDABE* reporter [2]. The ease of detecting bioluminescent signal from *luxCDABE* without the addition of substrates or inactivation of the bacterium makes this an ideal reporter for real time monitoring of bacteria and high throughput biology technologies.

*Yersinia pestis* causes the acute infection known as the plague. Human plague can manifest as three different forms. Bubonic plague arises in individuals who have been fed upon by an infected flea. The bacteria are regurgitated into the bite site by the flea and rapidly colonize the proximal lymph nodes. In these tissues, *Y. pestis* evades the immune system and replicates to high numbers. Without treatment, the bacteria can eventually colonize the bloodstream, leading to the development of septicemic plague. Cases of primary septicemic plague can also arise if *Y. pestis* is directly inoculated into the blood by the flea. From the blood, *Y. pestis* disseminates to other tissues in the host. Colonization of the lungs results in the development of pneumonia (called secondary pneumonic plague). Pneumonic plague patients can directly transmit *Y. pestis* to naïve individuals via contaminated aerosols, resulting in primary pneumonic plague [3], [4]. Direct aerosol transmission of *Y. pestis* has also raised concerns about the potential use of plague as a biological weapon [5].

Several examples of the use of bioreporters in *Yersinia* have been reported. Two independent high throughput screens for inhibitors of the *Yersinia* type III secretion system have used bioluminescent bioreporters. The first screen monitored changes in *yopE* transcription with a P*yopE*::*luxAB* reporter [6], while the second used a *lux* operon driven by a constitutive promoter to monitor bacterial growth [7]. Other groups have engineered *luxCDABE* reporters to be under the transcription control of promoters of virulence genes to monitor expression patterns of these genes [8]–[10]. In addition to these in vitro assays, a limited number of studies in *Yersinia* using bioluminescent reporters for optical imaging of whole animals have been reported. Trcek et al. developed an inducible *luxCDABE* reporter in *Y. enterocolitica* to monitor oral and IV infection [11]. The authors observed luminescent signal from the abdomen of live animals during oral infection, but due to the nature of the gastrointestinal tract, specific tissue localization required necropsy. However, whole animal imaging revealed unexpected colonization of the cervical lymph nodes that has been overlooked using conventional models. In *Y. pseudotuberculosis*, Thorslund et al. were able to differentiate infection by wild type (WT) or mutant bacteria using the pCD1-Xen4 reporter [12]. More recently, Nham et al. infected animals subcutaneously with WT *Y. pestis* harboring a plasmid-based luciferase reporter and demonstrated that bioluminescence could be used to localize bacteria to lymph nodes via whole animal imaging. They were also able to use bioluminescence to monitor the development of systemic disease [13].

Whole animal optical imaging has also been used to study pneumonic infection by several Select Agent pathogens. Independently, two groups demonstrated that experimental melioidosis could be visualized in the mouse model [14], [15]. Furthermore, Warawa et al. were able to visualize both upper and lower respiratory tract colonization, differentiate between colonization patterns of mutant bacteria, and show that luminescence detection from the thoracic cavity strongly correlated to bacterial numbers in the lung. Bina et al. developed a plasmid-based *luxCDABE* bioreporter in *Francisella tularensis* [16]. Using this system, they demonstrated that the volume of the bacterial suspension administered to mice could affect whether the bacteria were delivered to the lung [17]. These studies demonstrate the potential for use of bioluminescent-based optical imaging to monitor pneumonic plague.

Several animal models of human plague have been characterized to study *Y. pestis* pathogenesis and develop potential therapeutics [18]. Conventional models to study microbial pathogenesis use separate groups of animals to determine the survival of animals (e.g., LD_50_ and/or time to death analysis) or dissemination rate of the pathogen (by enumerating bacteria from specific tissues of subsets of animals sacrificed at various time points). In contrast, optical imaging models allow for temporal and spatial analysis of the infection and survival data to be acquired from the same animal. Potential advantages of optical imagining models are: 1) smaller number of animals required for studies, 2) ability to follow the course of the disease in the same animal over time, and 3) potential to identify unexpected dissemination routes.

Here we describe the development of two chromosomally-based *luxCDABE* reporters for use in *Y. pestis*. We demonstrate that these reporters can serve as sensitive bioreporters to monitor *Y. pestis* growth and survival under different conditions during in vitro growth. We also demonstrate that both bubonic and pneumonic plague infection can be monitored in live animals using these reporters via optical imaging. Finally, we show that the *luxCDABE* bioreporter can be used to compare and differentiate virulence phenotypes in animals without the need to sacrifice animals.

## Materials and Methods

### Bacterial strains, plasmids, and growth conditions

The bacterial strains and plasmids used in this study are listed in Table 1. *E. coli* was grown in Luria-Bertani (LB) broth at 37°C. *Y. pestis* was grown in Brain Heart Infusion (BHI) broth at 26 or 37°C (with 2.5 mM CaCl_2_). When appropriate, antibiotics were used at the following concentrations: kanamycin, 50 µg ml^−1^ (*E. coli*), 25 µg ml^−1^ (*Yersinia*); carbenicillin, 50 µg ml^−1^.

**Table 1:** Strains and plasmids used in this work.

*Bacterial Strains*		
MBLYP-001	*Y. pestis* CO92; one passage from YP003-1	[32]
MBLYP-043	MBLYP-001 with Lux_P*cysZK*_ reporter	This work
MBLYP-010	*Y. pestis* CO92 Δ*pla*; one passage from YP102	[27]
MBLYP-045	MBLYP-010 with Lux_P*cysZK*_ reporter	This work
YPA035	MBLYP-001 pCD1^(−)^	This work
YPA038	YPA035 with Lux_P*EM7*_ reporter	This work
YPA039	YPA035 with Lux_P*tolC*_ reporter	This work
YPA040	YPA035 with Lux_P*cysZK*_ reporter	This work
YPA047	YPA035 Δ*phoP*	This work
YPA073	YPA047 with Lux_P*EM7*_ reporter	This work
YPA048	YPA047 with Lux_P*tolC*_ reporter	This work
YPA049	YPA047 with Lux_P*cysZK*_ reporter	This work
YPA022	YPA035 with pGEN-*luxCDABE* plasmid	This work

*Plasmids*		
pGEN-*luxCDABE*	*Lux* operon with EM7 promoter	[20]
pUC18r6k-mini-Tn7(kanEW)	pUC18r6k-mini-Tn7 w/modified Kan cassette	[21]
pLOU027	pUC18r6k-mini-Tn7(kanEW):: Lux_P*EM7*_	This work
pLOU034	pUC18r6k-mini-Tn7(kanEW):: Lux_Pt*olC*_	This work
pLOU037	pUC18r6k-mini-Tn7(kanEW):: Lux_P*cysZK*_	This work
pTNS2	Tn7 transposase helper plasmid	[21]
pSKIPPY	pLH29 w/Cat cassette replaced with Kan cassette	[33]

The *Y. pestis phoP* mutant was generated using lambda red recombinase as previously described [19]. Briefly, regions flanking the *phoP* gene were amplified by PCR with primers DNA418 (5′-GAT TTC TAC ACC GTC GTG GG-3′) and DNA419 (5′-GAA GCA GCT CCA GCC TAC AC CAT ACA CCA ATC CTT GAT AAA ACG TTA AC-3′) for the 5′ fragment and primers DNA420 (5′-GGT CGA CGG ATC CCC GGA ATAG ACA CTA TGC TCA GAA AAA ATA ATA AAC CC-3′) and DNA421 (5′-GGT GAG TTG AGG TAA ACG AGA G-3′) for the 3′ region. The resulting products were gel purified and combined with a *kan* cassette flanked by FRT sites via overlapping extension PCR using primers DNA418 and DNA421. The resulting fragment was transformed into YPA035 expressing lambda red recombinase, followed by excision of the *kan* cassette, to generate YPA047.

The chromosomal *luxCDABE* reporters (Lux) were generated by first amplifying the *lux* operon, including the EM7 promoter, from pGEN-*luxCDABE* by PCR using primers DNA398 (5′-G GAG CTC CTC TGT CAT TTT CTG AAA CTC TTC ATG CTG-3′) and DNA399 (5′-G GAG CTC CCG CAT CAA CTA TCA AAC GCT TCG-3′) (engineered SacI restriction sites are underlined) [20]. The PCR product and pUC18r6k-mini-Tn7(kanEW) (a derivative of pUC18r6k-mini-Tn7 [21] in which the original *kan* cassette was replaced with the *kan* cassette from pKD13) were digested with SacI and ligated together to generate pLOU027. The EM7 promoter was subsequently removed from pLOU027 by digesting the plasmid with KpnI, which excised the promoter. The *tolC* promoter was amplified by PCR using primers DNA408 (5′-G GGT ACC GCC ACT CAT CGC AGT GTG-3′) and DNA409 (5′-G GGT ACC AGG ATC GTC AAA AAC CGA TAT AAG ACG-3′) and the *cysZK* promoter using primers DNA406 (5′-G GGT ACC ACT CTC GCC AAT ATT ATT GCG G-3′) and DNA407 (5′-G GGT ACC CGC CAA AAT ACG TCC GTT G-3′) (engineered KpnI restriction sites are underlined). PCR products were digested with KpnI and ligated into KpnI-digested pLOU027. Proper orientation of the promoters was confirmed by DNA sequencing. Reporters were integrated into the *Y. pestis* chromosome through site specific transposition as described previously to generate the Lux_PEM7_, Lux_P*tolC*_, and Lux_P*cysZK*_ bioreporter strains [21]. The antibiotic resistance cassette was excised from MBLYP-043 and MBLYP-045 as described previously [19].

To compare the sensitivity of the reporters, reporter strains YPA022, YPA038, YPA039, and YPA040 were inoculated in BHI broth in triplicate and grown for 15 hrs at 26°C. Serial 10-fold dilutions of the cultures were made in sterile 1× PBS, and the bacterial concentration of the dilutions was determined by enumerating on BHI agar. 100 µl aliquots were also transferred to a 96-well white plate and bioluminescence for each dilution was determined using a Synergy HT plate reader (BioTek, Winooski, VT) (1 sec read, sensitivity of 135). Linear regression analysis of the log transformed data was used to calculate the trend line, R^2^ values, and limit of detection.

To determine growth profiles and correlation between CFU and bioluminescence, YPA035, YPA038, YPA039, and YPA040 were grown for 15 hrs in BHI at 26°C. Bacteria were diluted into fresh medium to a concentration of 0.03 to 0.05 OD_600_/ml and grown for 12 hrs at either 26 or 37°C. Samples were harvested at various time points during growth to determine OD_600_, bioluminescence using a Synergy HT plate reader (1 sec read, sensitivity of 135), and bacterial numbers by serial dilution and enumeration on BHI agar. Linear regression analysis of the log transformed data was used to calculate the trend line and R^2^ values. To compare expression between 26 and 37°C, RLU per CFU was determined for each sample over the entire growth curve. Statistical significance was determined using the Mann-Whitney *t* test with a two-tailed nonparametric analysis.

### Survival of *Y. pestis* in the presence of antimicrobial compounds

To monitor survival of *Y. pestis* in antimicrobial compounds, YPA039 was grown for 15 hrs at 26°C. The OD_600_ of the culture was determined and bacteria were diluted to 1 OD_600_/ml. Bacteria were further diluted 100-fold in BHI to a final concentration of ∼10^6^ CFU/ml. 100 µl of bacteria were added to wells of a white 96-well plate. Bioluminescence for each well was determined with a Synergy HT plate reader (1 sec read, sensitivity of 135) to establish a baseline and then 100 µl of indicated dilutions of MicroChem-Plus (National Chemical Laboratories, Philadelphia, PA) or antibiotics (diluted in BHI) were added to each well. For MicroChem-Plus, the first reading was taken 2.5 mins after addition and every 1.3 mins thereafter until 14 mins. At 6 mins, a subset of samples was harvested, washed once with 1× PBS, and 10-fold serial dilutions of bacteria were spot plated on BHI agar. For antibiotics, the first reading was taken 10 mins after addition of antibiotics and every hr thereafter for 15 hrs. Plates were incubated at 26°C in the plate reader between reads. Samples were blanked against BHI only wells. At 4, 8, and 12 hrs, 100 µl of bacteria were harvested from each concentration and 10-fold serial dilutions were spot plated on BHI agar to determine CFU.

### Intracellular survival assays

RAW264.7 macrophages (ATCC, Manassas, VA) were seeded into white 96-well tissue culture plates and infected with 10^6^ CFU (MOI = 10) of the *Y. pestis* reporter strains YPA035, YPA038, YPA039, YPA040, YPA073, YPA048, or YPA049, as described previously [22]. Extracellular bacteria were killed by incubation with gentamicin (16 µg/ml) for 1 hr, followed by three washes with 1× PBS. Medium was replaced with DMEM+10% FBS containing 2 µg/ml gentamicin and plates were incubated at 37°C with 5% CO_2_ for 24 hrs. Bioluminescence was determined at various time points using a Synergy HT plate reader (1 sec reading, sensitivity of 135). For CFU determinations, cells were lysed with 1% Triton 100 and bacteria were enumerated by serial dilution and plating on BHI agar.

### *In vivo* imaging

All animal studies were approved by the University of Louisville Institutional Animal Care and Use Committee (protocol 10–117). Five- to 7-week-old female B6(Cg)-Tyr^c-2J^/J (albino C57Bl/6) mice (The Jackson Laboratory, Bar Harbor, ME) were maintained in the ABSL-3 vivarium with sterilized food and water *ad libitum* at the University of Louisville's Center for Predictive Medicine Regional Biocontainment Laboratory and imaging was performed in conjunction with the Center for Predictive Medicine BIO-Imaging Core. Hair was removed with clippers on the dorsal and ventral sides of the mice two days prior to infection. Mice were anesthetized using a ketamine-xylene mixture for infections and isoflurane for imaging. Mice were infected with MBLYP-043 (WT) or MBLYP-045 (Δ*pla*). For bubonic studies, mice were infected via injection of 200–400 CFU at the base of the tail or in the hind foot. For pneumonic infections, mice were infected via intranasal infection of 10^4^–10^5^ CFU. Beginning after infection, mice were monitored for disease symptoms twice daily and moribund mice were euthanized. For imaging, mice were anesthetized and images were taken using the IVIS Spectrum imaging system (Caliper Life Sciences, Hopkinton, MA). Average radiance (photons/sec/cm^2^) was calculated for regions of interest of infected animals and similar regions were analyzed from uninfected animals or tissues to determine background luminescence (used as the limit of detection). Statistical significance was determined using the Mann-Whitney *t* test with a two-tailed nonparametric analysis.

## Results

### Construction of a chromosomal luciferase reporter system in *Y. pestis*

**Figure 1:**
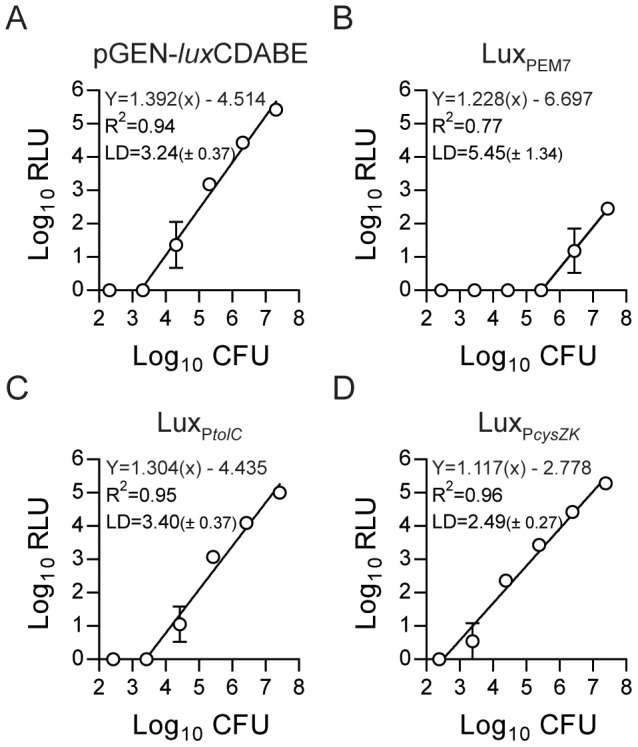
Sensitivities of chromosomal Lux reporters. The *luxCDABE* operon driven by different promoters was integrated into the *Y. pestis* chromosome using Tn7 transposition. Sensitivities of the Lux reporters were determined by making serial dilutions of the *Y. pestis* Lux strains (grown for 15 hrs) and determining the number of bacteria (CFU) and bioluminescence (RLU) in each dilution (n = 3). Linear regression analysis of the Log transformed data was used to calculate the trend line, R^2^ values, and the limit of detection [LD = Log_10_CFU (± standard deviation)]. (A) pGEN-*luxCDABE*, (B) Lux_PEM7_, (C) Lux_P*tolC*_, (D) Lux_P*cysZK*_.

**Figure 2:**
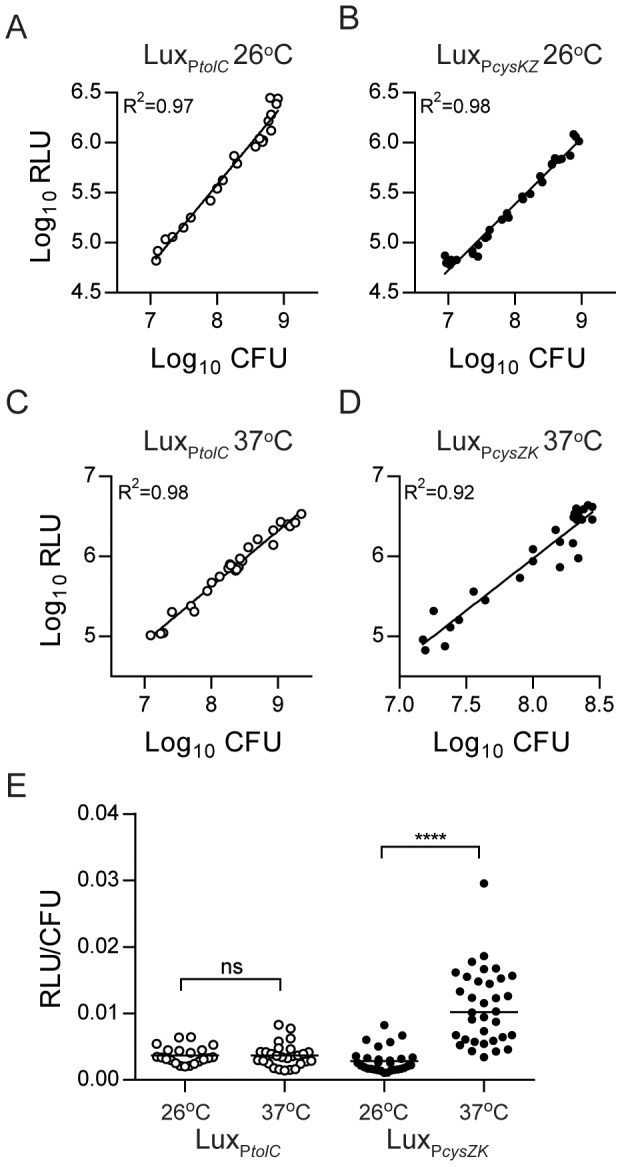
Correlation between bioluminescence and bacterial number. *Y. pestis* Lux_P*tolC*_ and Lux_P*cysZK*_ were diluted in BHI broth (n = 3) and grown at 26°C (A and B) or 37°C (C and D) for 12 hrs. Samples were harvested at multiple time points during growth to determine bioluminescence (RLU) and bacterial numbers (CFU). Linear regression analysis of the Log transformed data was used to calculate the trend line and R^2^ values. (E) To determine if temperature impacted expression of the Lux_P*tolC*_ (white circles) or Lux_P*cysZK*_ (black circles) reporters, we calculated the RLU/CFU for each sample in A–D and compared the ratios. Black bars represent median values and statistical significance was determined using the Mann-Whitney *t* test with a two-tailed nonparametric analysis (**** = p<0.0001, ns = not significantly different).

Our preliminary data demonstrated that in *Yersinia* a *luxCDABE* based-reporter was >200-fold more sensitive than equivalent fluorescent reporters using dsRED or EGFP (data not shown). Therefore, we developed a bioreporter using the *lux* operon in *Y. pestis*. Using a Tn7-based system, we integrated the entire *luxCDABE* operon driven by the EM7 promoter from pGEN-*luxCDABE* into the *Y. pestis* chromosome [20], [21]. Integration of the reporter into the chromosome greatly reduced the amount of bioluminescence produced per bacterium compared to *Y. pestis* with pGEN-*luxCDABE* (likely due to a decrease in copy number), resulting in an average limit of detection of 2.84×10^5^ CFU (range = 1.30×10^4^ to 6.23×10^6^ CFU) for the chromosomal reporter (Fig. 1A and B). To increase the sensitivity, we replaced the EM7 promoter with one of two different promoters. We selected the *tolC* promoter from *Burkholderia pseudomallei*, which was used in a similar reporter in *B. pseudomallei* [14], and the *cysZK* promoter from *Y. pestis*, which was identified as a strong constitutive *Y. pestis* promoter [23]. Expression of the luciferase operon from the *tolC* promoter increased the chromosomal reporter sensitivity by ∼100-fold (average limit of detection = 2.5×10^3^ CFU, range = 1.09×10^3^ to 5.86×10^3^ CFU) and approached the sensitivity of pGEN-*luxCDABE* (Fig. 1C). The *cysZK* promoter further increased the sensitivity by an additional 10-fold, establishing an average limit of detection of 3.06×10^2^ CFU (range = 1.08×10^2^ to 5.76×10^2^ CFU) (Fig. 1D). As reported by Bland et al., we also observed increased expression of P*cysZK* at 37°C, but importantly, the Lux_P*cysZK*_ strain maintained a direct correlation between bacterial numbers (CFU) and light production (RLU) during continuous growth at both temperatures (Fig. 2). Lux_P*tolC*_ activity did not appear to be influenced by temperature and maintained a strong direct correlation between CFU and RLU at both temperatures (Fig. 2).

**Figure 3:**
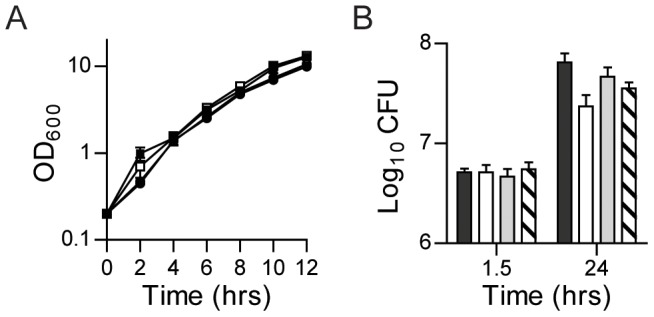
Lux reporters do not impact fitness of *Y. pestis*. To determine if carriage of the Lux reporters impacted *Y. pestis* fitness, (A) growth of the *Y. pestis* Lux bioreporter strains (n = 3), and (B) survival in macrophages (n = 3) were compared to WT *Y. pestis* without a Lux reporter. WT (no reporter) = • or black bar; Lux_PEM7_ = ○ or white bar; Lux_P*tolC*_ = □ or gray bar; Lux_P*cysZK*_ = ▪ or hatched bar.

To ensure that expression of the *lux* operon did not affect growth of *Y. pestis*, we determined the growth rate of the *Y. pestis* reporter strains in vitro (Fig. 3A). No significant differences were observed between WT *Y. pestis* (no reporter) or strains carrying the three chromosomal reporters. We further examined whether the Lux reporters impacted fitness of *Y. pestis* in the macrophage model. As seen in broth culture, the Lux reporters did not negatively impact the survival/replication of *Y. pestis* in macrophages, and we observed similar levels of replication by the reporter strains in RAW264.7 macrophages as WT *Y. pestis* without a reporter (Fig. 3B). Together these data demonstrate that integration of the *lux* operon driven by either P*tolC* or P*cysZK* generated a sensitive luciferase reporter that does not appear to impact *Y. pestis* growth and whose light production directly correlates to bacterial number.

### Using the *Y. pestis* Lux reporters as bioreporters

**Figure 4:**
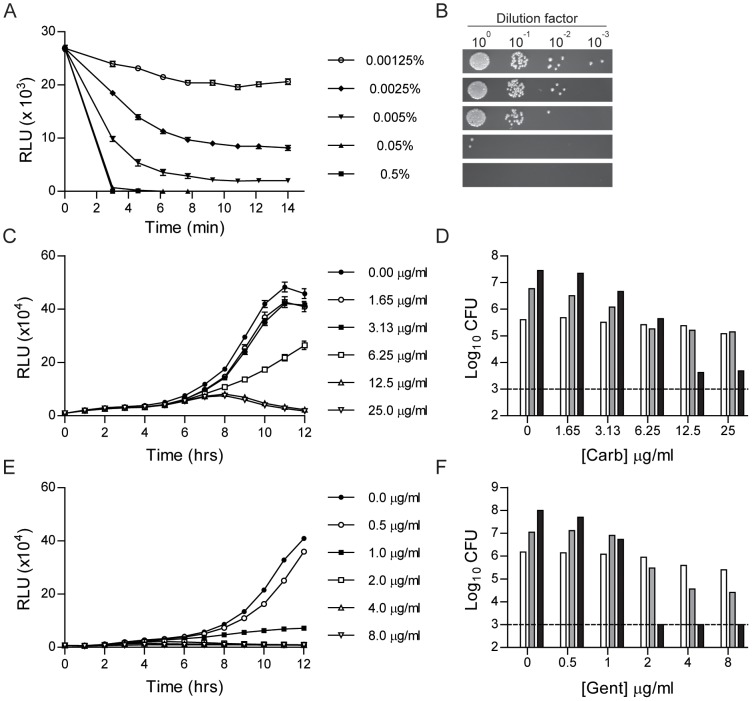
Use of Lux_P__*tolC*_ to monitor survival of *Y. pestis* in the presence of antimicrobial compounds. *Y. pestis* Lux_P*tolC*_ was incubated with increasing concentrations of antimicrobials (n = 9) in a 96-well format and bacterial survival was monitored by measuring bioluminescence. (A) Bioluminescence readings (RLU) from *Y. pestis* Lux_P*tolC*_ incubated with MicroChem-Plus for 14 mins. (B) At 6 mins during incubation with MicroChem-Plus, bacteria were harvested from a subset of wells, washed, serially diluted, and spot plated on agar to determine bacterial CFU. (C) Bioluminescence readings (RLU) from *Y. pestis* Lux_P*tolC*_ incubated with carbenicillin for 12 hrs. (D) At 4 (white), 8 (gray), and 12 (black) hrs during incubation with carbenicillin bacteria were harvested from a subset of wells to determine bacterial CFU. (E) Bioluminescence readings (RLU) from *Y. pestis* Lux_P*tolC*_ incubated with gentamicin for 12 hrs. (F) At 4 (white), 8 (gray), and 12 (black) hrs during incubation with gentamicin, bacteria were harvested from a subset of wells to determine bacterial CFU. For D and F, the dotted line represents the limit of detection.

Due to the requirement for a constant supply of O_2_, FMNH_2_, and aldehydes for the Lux system to produce light, bioluminescence only occurs in actively growing bacteria [2]. This property, in conjunction with the direct correlation between bioluminescence and bacterial numbers for the Lux_P*tolC*_ and Lux_P*cysZK*_ reporters, suggests that these reporters can be used to monitor *Y. pestis* survival in real time. To test this hypothesis, we incubated *Y. pestis* Lux_P*tolC*_ with decreasing concentrations of a chemical disinfectant (MicroChem-Plus), and then monitored bacterial survival as a function of bioluminescence (Fig. 4A). At 6 mins post-exposure, samples were harvested, washed and plated to determine if bioluminescence readings correlated with bacterial numbers (Fig. 4B). Within 2 mins of exposure to MicroChem-Plus at concentrations ≥0.05%, we were unable to detect bioluminescence from the *Y. pestis* cultures. This correlated with viable bacteria, as at these concentrations, viable bacteria were below the level of detection of the Lux_P*tolC*_ reporter. At levels of MicroChem-Plus <0.05% we observed a dose dependent reduction in bioluminescence that directly correlated to the number of bacteria recovered after six mins of incubation.

To further demonstrate that bioluminescence can differentiate bacteria survival, *Y. pestis* Lux_P*tolC*_ was incubated in 96-well plates with increasing concentrations of carbenicillin or gentamicin. Plates were incubated for 12 hrs at 26°C, and bioluminescence was detected every hr. These readings indicated a dose dependent bacterial growth inhibition, with lower bioluminescence readings observed as antibiotic concentrations increased (Fig. 4C and E). To confirm that bioluminescence readings correlated with bacterial numbers, a subset of samples was harvested at 4, 8, and 12 hrs and bacterial CFUs were determined by conventional enumeration (Fig. 4D and F). As seen for bioluminescence, we also observed a dose dependent response in bacterial CFU. Together these data demonstrate that bioluminescence can be used to monitor changes in bacterial survival.

### Differentiation between bacterial phenotypes in vitro using *Y. pestis* Lux bioreporters

**Figure 5:**
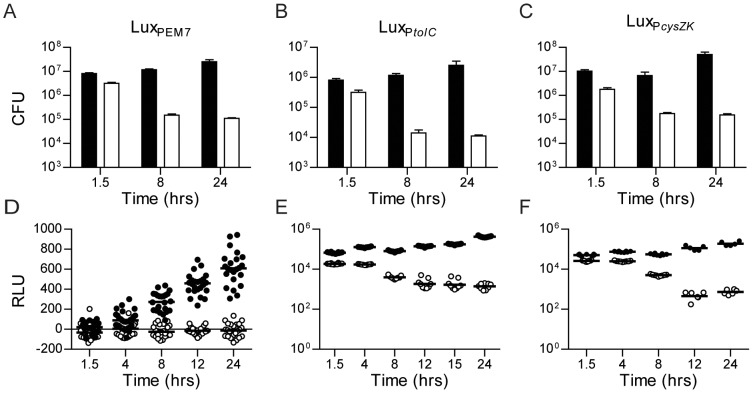
Survival of *Y. pestis* Lux reporters in macrophages. RAW264.7 macrophages were infected with *Y. pestis* Lux reporter strains, extracellular bacteria killed by gentamicin, and bacterial survival monitored by CFU determination (A–C) or bioluminescence (D–F). Data from WT *Y. pestis* is represented by black symbols and from an attenuated Δ*phoP* mutant by white symbols. (A and D) are strains with the Lux_PEM7_ reporter (n = 3 for CFU, n = 24 for RLU), (B and E) are strains with the Lux_P*tolC*_ reporter (n = 3 for CFU, n = 12 for RLU), and (C and F) are strains with the Lux_P*cysZK*_ reporter (n = 3 for CFU, n = 12 for RLU).

To further demonstrate that the *Y. pestis* Lux bioreporters can be used to monitor bacterial numbers in a biological system, we infected macrophages with WT *Y. pestis* pCD1(-) or a mutant defective in macrophage survival (Δ*phoP*) carrying our reporter constructs. RAW264.7 macrophages were infected with the reporter strains and extracellular bacteria were killed with gentamicin. At several time points post-infection, bioluminescence was measured using a plate reader. In addition, at 1.5, 8, and 24 hrs post-infection, samples were also harvested to determine bacterial numbers by conventional bacterial enumeration techniques. CFU data demonstrated that all three of the WT *Y. pestis* reporter strains survived within the macrophages, but the Δ*phoP* mutant strains were attenuated and bacterial numbers differed from WT by approximately two orders of magnitude over the course of the assay (Fig. 5A–C). The sensitivity of the bioluminescence signal produced by the Lux_P*tolC*_ and Lux_P*cysZK*_ reporter strains allowed for easy differentiation between WT and Δ*phoP* phenotypes (Fig. 5E–F). In contrast, the lower sensitivity of the Lux_PEM7_ reporter made it more difficult to differentiate the Δ*phoP* phenotype (Fig. 5D). While RLU data from the WT Lux_PEM7_ strain correlated with CFU data, the bioluminescent signal of the Δ*phoP* Lux_PEM7_ strain quickly dropped below the limit of detection of the reporter, resulting in a loss of correlation between bacterial CFU and RLU for this assay. These data demonstrate that the Lux_P*tolC*_ and Lux_P*cysZK*_ bioreporters can be used to monitor changes in bacterial populations in biological systems in vitro.

### In vivo imaging of bubonic plague

**Figure 6:**
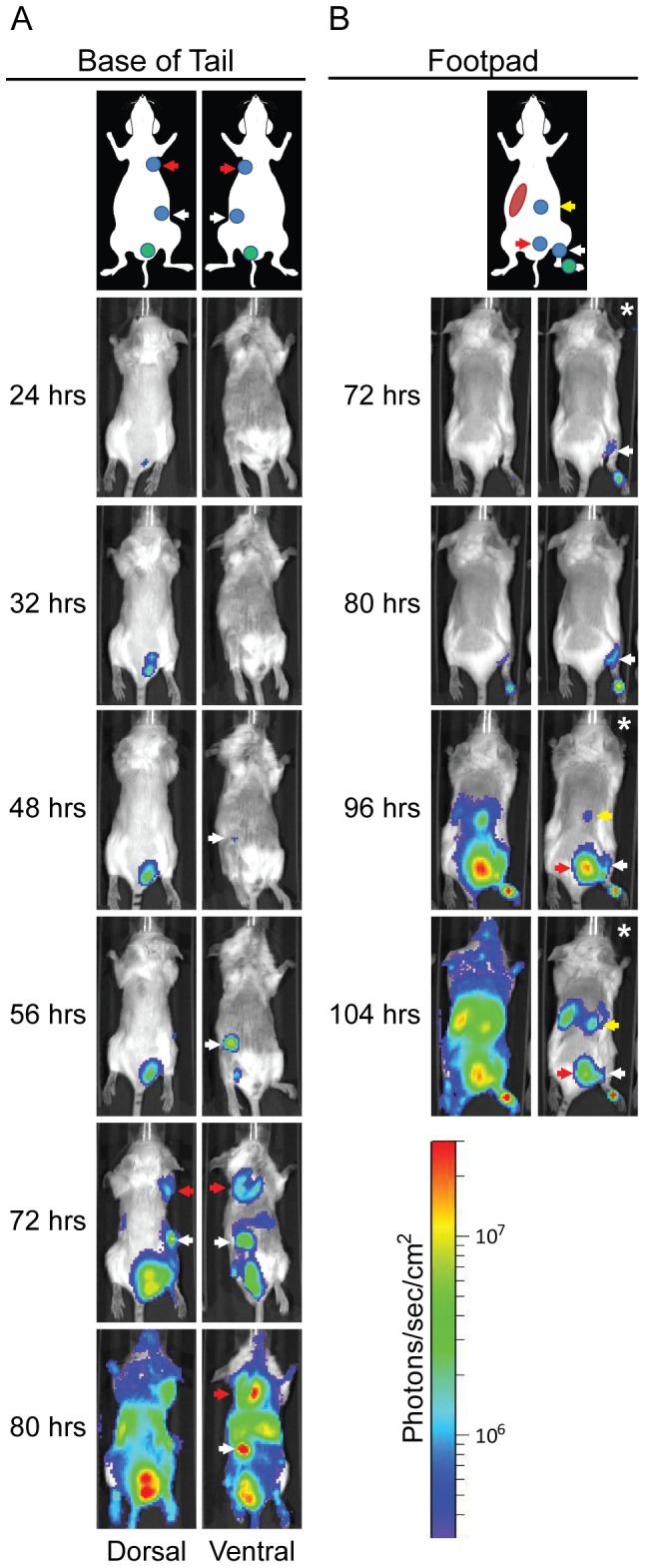
Dissemination of *Y. pestis* during bubonic infection. Mice were infected with ∼200 CFU of *Y. pestis* Lux_P*cysZK*_ subcutaneously at the base of the tail (A) or in the footpad (B) and imaged using an IVIS Spectrum. The lymph node drainage basin for each inoculation site is diagrammed above the images [24], [25]. Location of the inoculation site is shown as a green circle, lymph nodes as blue circles, and the spleen as a red oval. For (A), the white arrow denotes the subiliac LN and the red arrow the axillary LN. For (B), the white arrow denotes the popliteal LN, the red arrow the sciatic LN, and the yellow arrow the renal LN. All images were adjusted to the radiance scale shown, except for the images in (B) marked with * in upper right corners. For these each image was adjusted to a different radiance to allow for visualization of specific tissues.

**Figure 7:**
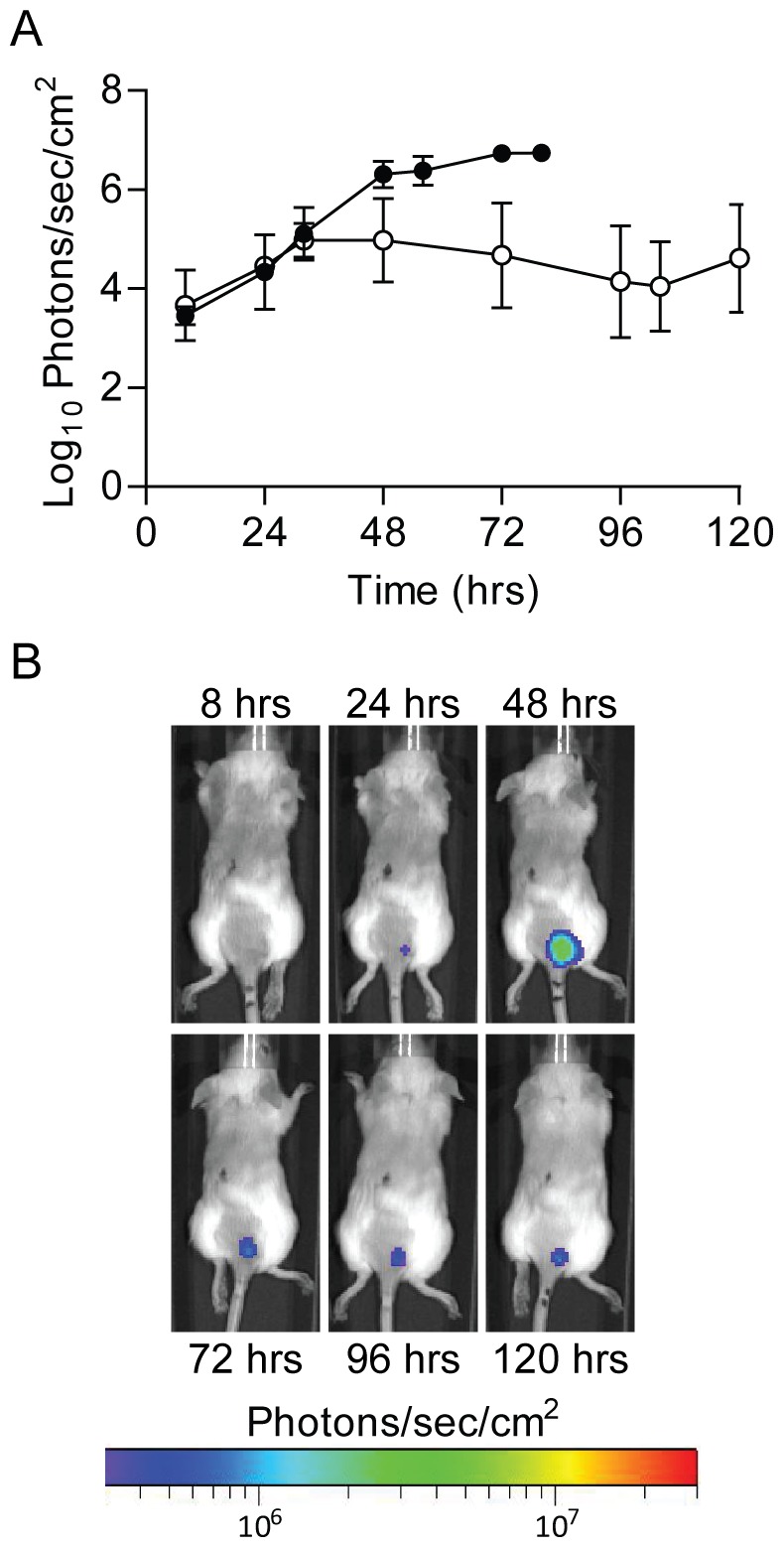
Continued bioluminescence from inoculation site. Mice were infected with ∼200 CFU of WT (n = 5) or Δ*pla* (n = 5) *Y. pestis* Lux_P*cysZK*_ subcutaneously at the base of the tail and imaged using an IVIS Spectrum. (A) The average bioluminescence detected from the inoculation site was determined over the course of the infection. Black and white symbols represent animals infected with WT or Δ*pla Y. pestis*, respectively. (B) Sequential images from a representative animal infected with Δ*pla Y. pestis* Lux_P*cysZK*_.

The high sensitivity of the Lux_P*cysZK*_ bioreporter that we observed in vitro suggested that it could also be used to monitor plague infection in vivo. Bubonic plague is the most common form of human plague and results from flea transmission. In the laboratory, bubonic plague can be modeled by intradermal or subcutaneous inoculation of mice with *Y. pestis*. After inoculation, the bacteria disseminate to the draining lymph node. Eventually the bacteria enter into the bloodstream to cause a systemic infection. To determine if the Lux_P*cysZK*_ bioreporter could be used to monitor bubonic infection, specifically lymph node colonization, mice were challenged with the WT CO92 Lux_P*cysZK*_ strain, and infection was monitored using whole animal optical imaging (Fig. 6). Mice were inoculated at the base of the tail with approximately 200–400 CFU of the bioreporter strain. The sensitivity of the bioreporter strain allowed us to detect bioluminescent signal from the inoculation site as early as 8 hrs post-inoculation. Furthermore, signal increased over time at the inoculation site, indicating that *Y. pestis* survives and replicates at the inoculation site over the course of the infection (Fig. 7A).

Previous work has defined the lymphatic drainage basin for the base of the tail to be the subiliac (also referred to as the inguinal) and the axillary lymph nodes (LN) [24], [25]. We began to detect luminescent signal from the subiliac LN starting between 48 and 72 hrs post-inoculation (Fig. 6A, white arrows). Approximately 8–15 hrs after first detection in the subiliac LN, signal began to be detected in the axillary LN, indicating bacterial dissemination to these nodes (Fig. 6A, red arrows). For both lymph nodes, the bioluminescent signal continued to increase in the tissues over the course of the infection, indicating bacterial proliferation. By 72 hrs post-inoculation, we began to detect bioluminescence from other regions, indicating systemic infection. The animals succumbed to infection by 96 hrs post-inoculation.

To further demonstrate that our bioreporter can be used to monitor bubonic plague dissemination, an additional group of mice was infected in the footpad with the WT CO92 Lux_P*cysZK*_ strain. Previous work has demonstrated that dyes can disseminate from this site via two different drainage basins in mice [24], [25]. The first basin drains to the popliteal LN, followed by the sciatic and renal LNs. Alternatively, drainage to the same basin as from the base of the tail can occur. In these studies we observed *Y. pestis* disseminating only through the former drainage basin from the footpad (Fig. 6B). Bioluminescent signal was first detected in the popliteal LN at about 72 hrs post-inoculation. Signal was detected 24 hrs later from regions corresponding to the sciatic and renal LNs. At this time we also were able to detect signal from the spleen. Together these data demonstrate that lymph node colonization and dissemination of *Y. pestis* can be tracked in live animals via optical imaging using the Lux_P*cysZK*_ bioreporter.

### *In vivo* imaging of pneumonic plague

**Figure 8:**
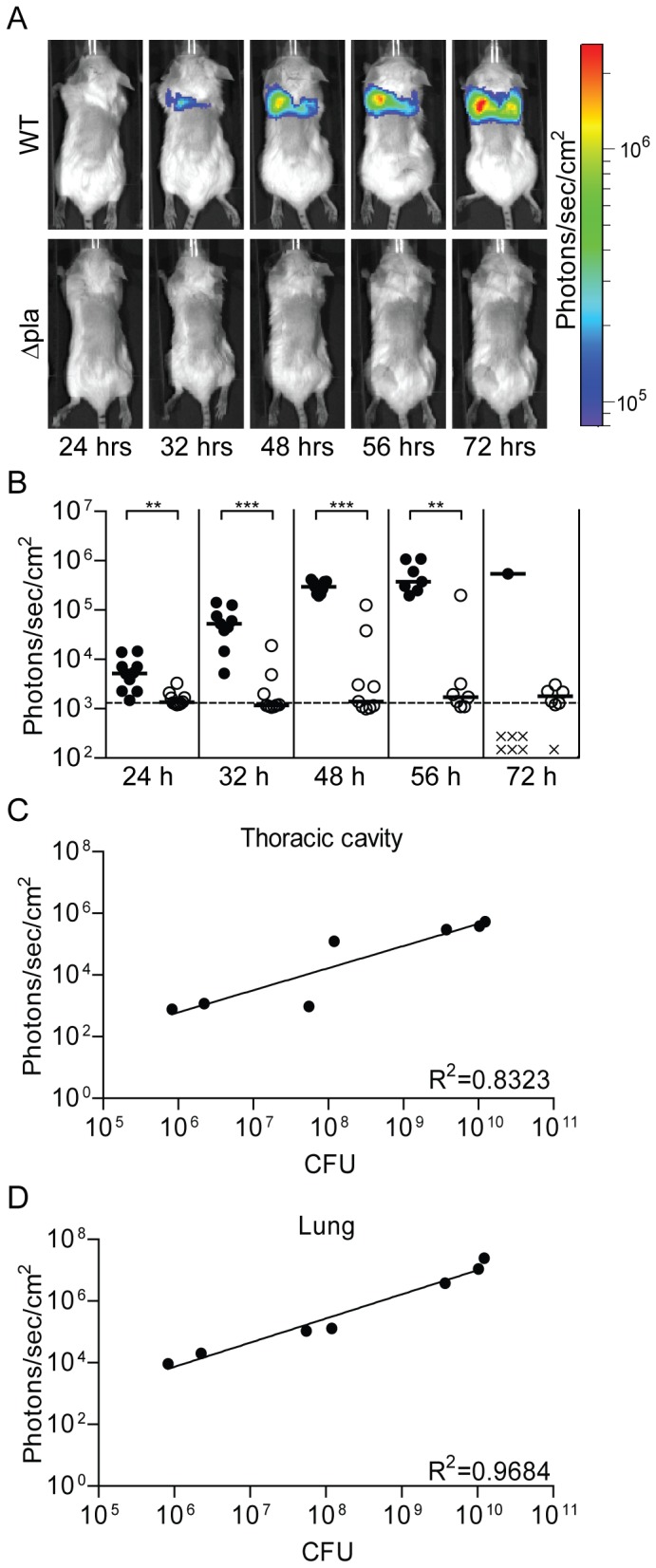
Progression of pneumonic infection. Mice were infected with 5×10^4^–1×10^5^ CFU of *Y. pestis* Lux_P*cysZK*_ intranasally and imaged using an IVIS Spectrum. (A) Sequential images from representative animals. (B) For each animal, average bioluminescence was calculated for the thoracic cavity using the ROI tool in Living Image 3.2 software package. Black and white symbols represent animals infected with WT or Δ*pla Y. pestis*, respectively. Dotted line represents the limit of detection based on images from uninfected animals. ** = p<0.005, *** = p<0.001. At various time points, lungs were harvested from a subset of animals to determine bacterial loads (CFU) and compared to bioluminescence from the thoracic cavity (C) or from the lungs ex vivo (D).

Primary pneumonic plague occurs when aerosols containing *Y. pestis* are inhaled by a naïve individual. This form of disease can also be modeled in the mouse using the intranasal route of infection [26]. To determine if the Lux_P*cysZK*_ bioreporter can be used to monitor pneumonic infection, we challenged mice intranasally with the WT CO92 Lux_P*cysZK*_ strain and followed the progression of pneumonic plague by optical imaging. Bioluminescent signal could be detected from the thoracic cavity of all mice as early as 24 hrs post-inoculation and increased throughout the course of infection (Fig. 8A and B). To demonstrate that the bioluminescence signal directly correlated with bacterial numbers, lungs were harvested from a subset of animals after the 24, 48, and 72 hrs imaging sessions. The tissues were imaged and bacterial numbers in the lungs were determined. Bioluminescent signal from imaging of the thoracic cavity directly correlated to lung CFU (Fig. 8C; R^2^ = 0.8323). The significance of the correlation increased further when comparing signal directly from harvested lungs to CFU (Fig. 8D; R^2^ = 0.9684). Animals infected with the Lux_P*cysZK*_ strain succumbed to infection between 60 and 80 hrs post-infection, a similar time to death as seen for *Y. pestis* without a reporter [26], [27].

### Differences in phenotypes can be detected *in vivo* using the Lux_P*cysZK*_ bioreporter

To demonstrate that whole animal imaging using the Lux_P*cysZK*_ bioreporter can differentiate between virulence phenotypes, we transferred the reporter into a *Y. pestis* Δ*pla* mutant. Pla is required for the development of bubonic plague, and a *pla* mutant is unable to disseminate from the inoculation site to the draining LN [28]–[30]. In the bubonic model, we observed bioluminescent signal from the inoculation site of *Y. pestis* Δ*pla* Lux_P*cysZK*_ infected animals as early as 8 hrs post-infection (Fig. 7A). Signal increased at the inoculation site at a rate comparable to WT infected animals until 36 hrs post-infection. After 36 hrs, signal from WT infected animals continued to increase, but the signal from Δ*pla* infected animals plateaued, remaining about 1–2 logs lower than WT signal for the remainder of the experiment. No signal was observed from the draining LN from Δ*pla* infected animals (Fig. 7B), supporting previous data that the mutant is unable to disseminate to the LN after intradermal infection [30]. However, one Δ*pla* infected animal (n = 9) appeared to develop primary septicemic plague, as no signal was detected from the lymph nodes prior to systemic signal (data not shown).

**Figure 9:**
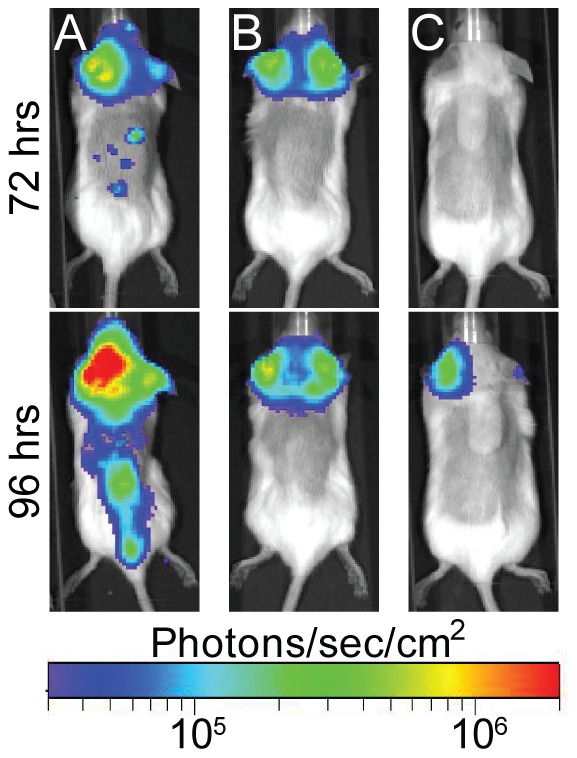
Extended imaging of animals intranasally infected with Δ*pla*. 30% of animals infected intranasally with the Δ*pla* mutant in Figure 6 developed bioluminescence signal from regions corresponding to the head. A, B, and C represent three individual animals. Animal A also represents an example of a Δ*pla* infected animal that developed septicemic plague.

In the model for pneumonic plague, the Δ*pla* mutant colonizes the lungs but is unable to proliferate in these tissues [27]. As expected, we observed low levels of bioluminescence from the thoracic cavity of mice infected intranasally with *Y. pestis* Δ*pla* Lux_P*cysZK*_, correlating with low levels of bacteria in these tissues (Fig. 8). Importantly, compared to WT infected mice, luminescence from the Δ*pla* infected animals was significantly lower at all time points, except at the 72 hr time point when there were not enough WT animals to calculate significance (Fig. 8B). While the Δ*pla* mutant does not proliferate within the lungs during pneumonic infection, the LD_50_ of the mutant is similar to WT *Y. pestis*, likely due to the development of septicemic plague [27]. The sensitivity of the Lux_P*cysZK*_ bioreporter allowed us to observe the development of septicemic plague in Δ*pla* infected animals (Fig. 9A). Furthermore, as we monitored the Δ*pla* infected animals, we also observed that a subset of animals developed bioluminescent signal near the ears which we did not observe in WT infected animals (Fig. 9). Together these data demonstrate that whole animal imaging with the Lux_P*cysZK*_ bioreporter can differentiate between bacterial phenotypes during both bubonic and pneumonic plague infection.

## Discussion

The bacterial *luxCDABE* operon produces a bioluminescent signal that can be used as a bioreporter to monitor bacterial numbers in real time. We developed two *luxCDABE* reporters for use in *Y. pestis* to monitor bacterial survival. We demonstrated that these reporters can be used to monitor bacterial numbers in the presence of antimicrobial compounds, during intracellular infection, and in animal models for plague infection. Unlike plasmid-based systems previously used in *Yersinia* spp. [9], [11]–[13], these reporters are integrated into the chromosome. A chromosomal-based system has several characteristics that may be advantageous for future applications. First, integration of the reporter into the chromosome does not require antibiotic selection for maintenance and will likely be more stable than a plasmid-based system. Second, while plasmid reporters may be maintained without antibiotics for a period of time, especially with integrated toxin-anti-toxin maintenance mechanisms [13], [31], the plasmid still confers resistance for the selectable marker carried by the plasmid. Consequently, that marker is not available for further use (for example, to maintain other plasmids). The chromosomal reporters described here were engineered using a system that allows for the antibiotic marker to be removed after integration [21]. Therefore, the marker (in this case Kan) can be reused in downstream applications.

One advantage of a plasmid-based reporter system is that plasmids are often maintained at increased copy numbers compared to the chromosome, which can increase the sensitivity of the reporter. In fact, we observed a dramatic decrease in sensitivity when we moved the *luxCDABE* operon from a plasmid to the chromosome. To overcome this problem we removed the promoter from the original construct and replaced it with a promoter we hypothesized would increase the expression of the *lux* operon. We chose two different promoters to test. The first promoter was from *B. pseudomallei* (P*tolC*) and had been used to successfully develop a similar chromosomal reporter for this bacterium [14]. This promoter increased the sensitivity to the levels of the original plasmid-based reporter. The second promoter was originally identified by Bland et al. as being a strong constitutive promoter in *Y. pestis* (P*cysZK*) [23]. This promoter further increased the sensitivity to a level approximately 10-fold higher than the Lux_P*tolC*_ or pGEN-*luxCDABE*. Importantly, we saw no deleterious impact of increased *luxCDABE* expression from our reporters on *Y. pestis* fitness during growth in vitro, in cell culture, or in the animal models. Therefore, we successfully engineered a chromosomal luciferase reporter that is 10-fold more sensitive than a widely used plasmid-based reporter, without attenuating growth of *Y. pestis*.

For both the Lux_P*tolC*_ and Lux_P*cysZK*_ reporters we observed a direct correlation between bioluminescence and *Y. pestis* numbers. This characteristic is important and demonstrates that bioluminescence readings from these reporters can be used to quantify bacterial numbers. Furthermore, the sensitivity of the reporter and easy detection methods allow these bioreporters to be used in large scale formats. For example, we demonstrated that we could use the Lux_P*tolC*_ bioreporter to monitor bacterial growth in a 96-well format in the presence of antimicrobial compounds. Using this format we were able to easily determine the MIC for both carbenicillin and gentamicin. Furthermore, because we could monitor the bacteria in real time, we were also able to observe differences in growth patterns of *Y. pestis* in these two antibiotics. For example, *Y. pestis* incubated in inhibitory concentrations of carbenicillin (12.5 and 25 µg/ml) did not begin to decrease in bioluminescence until after 8 hrs into the assay, indicating that while bacterial growth might be inhibited, the bacteria were not killed by the antibiotic until after that time (Fig. 4C). In contrast, bioluminescence signal from bacteria incubated with inhibitory concentrations of gentamicin (2, 4, and 8 µg/ml) steadily decreased over the course of the assay, suggesting that bacterial death occurred much earlier (Fig. 4E). These hypotheses are supported by the CFU data that demonstrated that bacterial numbers did not begin to decrease in the carbenicillin samples until between 8 and 12 hrs, compared to between 4 and 8 hrs in gentamicin samples (Fig. 4D and F). These phenotypes can be explained by the mechanisms of action of the two antibiotics. Gentamicin blocks protein synthesis and quickly inhibits bacterial growth, whereas carbenicillin targets the bacterial peptidoglycan, which over time weakens the cell wall, leading to osmotic lysis, but allows for a short period of proliferation. The sensitivity and correlation between bioluminescence and bacterial numbers indicate that the bioreporters can be used to monitor *Y. pestis* survival in high throughput screens for new anti-*Y. pestis* compounds.

While we saw a consistent correlation between bioluminescence and bacterial CFU in all of the assays we reported, macrophages infected with Δ*phoP* Lux_P*cysZK*_ demonstrated a decrease in bioluminescence between 8 and 24 hrs without a significant difference in CFU between these two time points. The same phenotype was not observed in the WT Lux_P*cysZK*_ strain or in the Lux_P*tolC*_ strains, all of which maintained correlation between RLU and CFU (Fig. 5). These observations demonstrate that depending on the specific experimental assay, one bioreporter may more accurately represent bacterial numbers than the other. Furthermore, while the *Y. pestis* Lux_P*cysZK*_ bioreporter was more sensitive than the Lux_P*tolC*_ bioreporter in our initial studies (Fig. 1), sensitivities of the bioreporters may change under different experimental conditions. For example, we observed that Lux_P*cysZK*_ is more active at 37°C than 26°C. Therefore, optimization and validation of the bioreporters must be performed for each new assay as it is being developed.

Nham et al. recently reported the use of a plasmid-based bioluminescent bioreporter to follow the progression of bubonic plague in mice [13]. Using this bioreporter they demonstrated that spread of *Y. pestis* to the draining lymph nodes could be visualized in live animals via optical imaging. Furthermore, the authors were able to identify spread to the liver and spleen during disseminated (septicemic) plague. Similarly, we demonstrate here that the Lux_P*cysZK*_ bioreporter could be used in optical imaging of bubonic infection. The sensitivity of the Lux_P*cysZK*_ bioreporter allowed detection of bacteria at the inoculation site as early as 8 hrs post-infection, and we observed distinct dissemination patterns of *Y. pestis* Lux_P*cysZK*_ from two different inoculation sites that followed the predicted lymphatic drainage basins. As the infection progressed, we were able to identify the transition to systemic infection when bioluminescence was detected from the spleen. Eventually bioluminescence was detected from more peripheral sites, such as the feet and tail, demonstrating that bacterial concentrations reached levels in the blood that could be detected by optical imaging prior to the animals succumbing to infection.

Our data also demonstrate that WT bacteria are not cleared from the inoculation site over the course of the infection, and continuous increase of bioluminescence at the site indicates that the bacteria proliferate. It is still unclear whether secondary septicemic plague initiates from bacteria disseminating from the lymph nodes or the inoculation site, but our data suggest that viable bacteria remain at the inoculation site as a possible reservoir for septicemic spread. Interestingly, while the Δ*pla* mutant did not appear to proliferate to WT levels at the inoculation site, we continued to detect bioluminescent signal from this site for as long as 14 days post-inoculation (unpublished data). These data indicate that the mutant can survive at the inoculation site for an extended period of time, but survival at this site was not sufficient to lead to septicemic plague. However, one of the nine animals infected with the Δ*pla* mutant developed septicemic plague during our studies. The lack of detectable signal from the draining lymph nodes suggests that the bacteria disseminated into the bloodstream without first colonizing the lymph nodes. A similar rate of septicemic infection by the Δ*pla* mutant was previously reported by Sebbane et al. [30]. While these data may suggest septicemic plague arises from the inoculation site, we agree with Sebbane et al. that it is more likely that sepsis resulted from direct inoculation of the bacteria into the bloodstream during the infection and not from escape from the inoculation site. Additional studies are needed to further understand the dissemination of *Y. pestis* into the bloodstream.

In addition to bubonic infection, we also demonstrate that the Lux_P*cysZK*_ bioreporter is sensitive enough to monitor infection of deeper tissues colonized during pneumonic plague. Importantly, through enumeration of bacterial CFU in the lungs, we demonstrated that bioluminescence from the thoracic cavity directly correlates to bacterial numbers in the lungs. This correlation supports the use of bioluminescence to estimate bacterial burden in the lungs. Furthermore, we were easily able to differentiate between WT and Δ*pla* infected animals, suggesting that this bioreporter can be used to differentiate between mutant phenotypes in the animal. The ability to monitor the entire progression of plague in an individual animal via optical imaging allows for dissemination kinetics and survival data to be obtained from the same group of animals, resulting in smaller number of animals per experiment. Furthermore, optical imaging of plague with the Lux_P*cysZK*_ bioreporter may benefit therapeutic research, as it will allow researchers to observe the resolution of an established infection after treatment is initiated.

Optical imaging with the Lux_P*cysZK*_ bioreporter will also allow researchers to identify unexpected dissemination patterns that might be missed in conventional models. For example, in a subset of animals intranasally infected with the Δ*pla* mutant, we observed bioluminescence from a region near the ears, which we did not observe in WT infected animals. The precise tissues infected in these animals have yet to be identified, but colonization of tissues in this region would not have been detected without the whole animal imaging data. These data raise the possibility that systemic infection by the Δ*pla* mutant may arise from colonization of the upper respiratory tract as opposed to dissemination directly from the lungs. However, additional experiments to determine the frequency of this phenotype, correlation to systemic infection, and identity of infected tissues are required to test this hypothesis.
